# Multipartite entanglement indicators based on monogamy relations of *n*-qubit symmetric states

**DOI:** 10.1038/srep20302

**Published:** 2016-02-04

**Authors:** Feng Liu, Fei Gao, Su-Juan Qin, Shu-Cui Xie, Qiao-Yan Wen

**Affiliations:** 1 State Key Laboratory of Networking and Switching Technology, Beijing University of Posts and Telecommunications, Beijing, 100876, China; 2 School of Mathematics and Statistics Science, Ludong University, Yantai, 264025, China; 3 School of Science, Xi’an University of Posts and Telecommunications, Xi’an, 710121, China

## Abstract

Constructed from Bai-Xu-Wang-class monogamy relations, multipartite entanglement indicators can detect the entanglement not stored in pairs of the focus particle and the other subset of particles. We investigate the *k*-partite entanglement indicators related to the *α*th power of entanglement of formation (*α*EoF) for *k* ≤ *n*, ***α***ϵ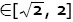 and *n*-qubit symmetric states. We then show that (1) The indicator based on *α*EoF is a monotonically increasing function of *k*. (2) When *n* is large enough, the indicator based on *α*EoF is a monotonically decreasing function of *α*, and then the *n*-partite indicator based on 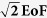 works best. However, the indicator based on 2 EoF works better when *n* is small enough.

Quantum correlations that comprise and go beyond entanglement are not monogamous. Only entanglement can be strictly monogamous^1^, that is, they obey strong constraints on how they can be shared among multipartite systems. This is one of the most important properties for multipartite quantum systems^2^. So these monogamy relations can be used to characterize the entanglement structure in multipartite systems^3^, and concretely *the difference between the left- and right-hand side of them can be defined as indicators* to detect multipartite entanglement not stored in pairs of the focus particle (e.g., the first particle) and the other subset of particles^4^.

For the squared concurrence, the indicator named three-tangle^3^ can be used to detect *genuine multipartite entanglement* (*which are entangled states being not decomposable into convex combinations of states separable across any partition*) in three-qubit pure states. However, for three-qubit mixed states, there exist some entangled states that have neither two-qubit concurrence nor three-tangle^5^. To reveal this critical entanglement structure, some multipartite entanglement indicators based on Bai-Xu-Wang-class monogamy relations for the entanglement of formation (EoF) have been proposed^4^,^6^,^7^. In this paper, we will study which multipartite entanglement indicator for EoF works better. By “work better” we mean that is larger than the other^8^.

We resolve the above problem in the following ways. Firstly, we prove that the *α*th power of EoF (*α*EoF, 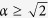) obeys a set of hierarchy *k*-partite 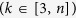 monogamy relations of Eq. (10) in an arbitrary *n*-qubit state 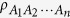. Here, the *k*-partition means the partition *A*_1_, 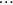, *A*_*k*−1_ and 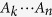. Based on these monogamy relations, a set of new multipartite entanglement indicators are presented correspondingly, which can work better than the 2 EoF-based indicators in *n*-qubit symmetric states. However, we find that the 2 EoF-based indicator can work better than the *α*EoF-based indicators for 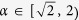 when *n* is small enough (e.g., *n* ≤ 9).

## Results

This section is organized as follows. In the first subsection, we review the monogamy relations for 2 EoF in *n*-qubit systems. We then prove in the second subsection that the *α*EoF obeys hierarchy *k*-partite monogamy relations for 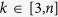 and any *n*-qubit states. In the third subsection, we construct the entanglement indicators on *n*-qubit symmetric states, and show their monotonic properties. Two examples are given in the forth subsection to verify these results.

### Review of monogamy relations for EoF

Coffman, Kundu, and Wootters^3^ proved the first monogamy relation for the squared concurrence in three-qubit states. Then, Osborne and Verstraete^9^ proved a set of hierarchy *k*-partite monogamy relations for the squared concurrence in *n*-qubit states 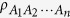, which have the form


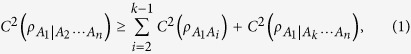


where *A*_1_ is the focus qubit, 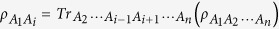, 
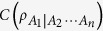
 is the concurrence of 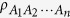 in the bipartition 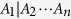, and 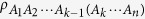 is a *k*-partite *n*-qubit state.

Based on these Osborne-Verstraete-class hierarchical monogamy relations in Eq. (1), a set of multipartite entanglement indicators can be constructed as follows


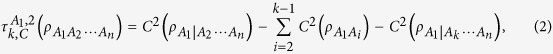


where the entanglement measure is the squared concurrence. These indicators can detect the entanglement not stored in pairs of *A*_1_ and any other *k* − 1 party (i.e., *A*_2_, 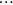, *A*_*k*−1_ and 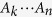)^4^. However, there exists a special kind of entangled state^10^ which has zero entanglement indicator. Moreover, the calculation of multiqubit concurrence is extremely hard due to the convex roof extension. Therefore, it is natural to ask whether other monogamy relations beyond the squared concurrence exist.

Recently, Bai *et al.*^4^ and Oliveira *et al.*^11^ respectively proved that 2 EoF is monogamous in *n*-qubit states, as follows


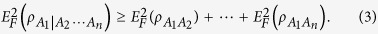


Moreover, Bai *et al.*^6^ exactly showed that there are a set of hierarchy *k*-partite monogamy relations for 2 EoF in an arbitrary *n*-qubit states, which obey the relation


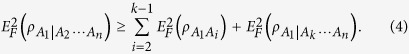


Generally, Zhu and Fei^7^ proved that *α*EoF obeys the following monogamy relation in *n*-qubit states,


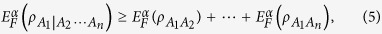


where 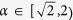. (In fact, Eq. (5) obviously satisfies for *α* > 2 which can be obtained from Eq. (4) and ref. 12.)

Because some bipartite multiqubit EoF of 
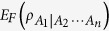
 can be calculated via quantum discord^13^,^14^, the entanglement indicator 
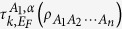
 from Eqs (3, 4, 5) can be obtained and can characterize multipartite entangled states in some *n*-qubit states^4^,^6^,^7^. In these entanglement indicators, how to choose a better indicator to detect that there exists multipartite entanglement is a problem. In the following subsections, we will try to resolve the problem.

### Hierarchy *k*-partite monogamy relations for *α*EoF

In this subsection, we firstly summary of some existing conclusions, and then get the hierarchy *k*-partite monogamy relations for *α*EoF.

As we know, EoF is a well defined measure of entanglement for bipartite states. For any two-qubit state *ρ*_*AB*_, an analytical formula was given by Wootters^15^ as follows


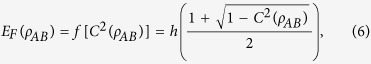


where 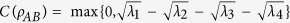 is the concurrence with the decreasing nonnegative *λ*_*i*_ being the eigenvalues of the matrix 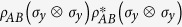. Here, 
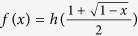
, and 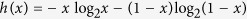 is the binary Shannon entropy. Recently, Bai *et al.*^6^ proved that *f*(*x*) is a monotonic and concave function of *x*. Moreover, Zhu and Fei^7^ proved that *f*(*x*) satisfies the following relation


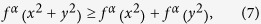


where 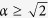, *x* and 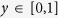. They also proved that EoF obeys the following relation


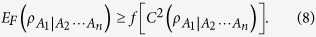


for the bipartite quantum state 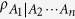 in 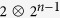 systems. Because a 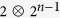 pure state 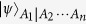 is equivalent to a two-qubit state under the Schmidt decomposition^16^, we have


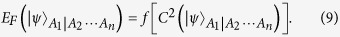


From Eqs (1) and (6, 7, 8, 9) for *n*-qubit systems, we can easily obtain that the following hierarchy *k*-partite monogamy relation holds.

**Theorem 1** For any *n*-qubit state 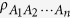, EoF satisfies the following monogamy relation


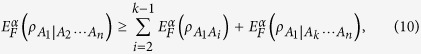


where 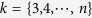 and 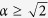.

The *α*EoF satisfies the hierarchy monogamy inequality (10) for any 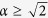, while the *α*th power of concurrence satisfies hierarchy monogamy inequalities for any *α* ≥ 2^9^,^12^. This phenomenon shows a difference between the two kinds of entanglement measures. On the other hand, the inequality (10) is a generalization of Eq. (5) in ref. 6 and Eq. (19) in ref. 7. More specifically, Eq. (10) equals to Eq. (4) when *α* = 2, and is the same as Eq. (5) when *k* = *n*.

### Properties of hierarchy entanglement indicators

For any *n*-qubit state 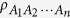 and *α*EoF 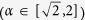, we can define a hierarchy entanglement indicator based on the corresponding monogamy relation in Eq. (10) as follows


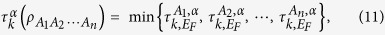


where


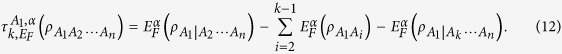


It can be used to detect the entanglement for the *k*-partite case of an *n*-qubit system^6^ not stored in pairs of *A*_1_ and any other *k* − 1 party.

Here it should be noted that, different from the hierarchy entanglement indicator of the concurrence, the indicator of EoF depends on which qubit is chosen to be the focus qubit. Fortunately, the indicators of the concurrence and EoF are all focus-independent in symmetric quantum systems. In the following, we give some properties about the indicators of EoF only for *n*-qubit symmetric states.

**Theorem 2** For any *n*-qubit symmetric state 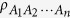, the hierarchy entanglement indicator satisfies


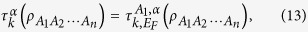


and it is a monotonically increasing function of *k*, where 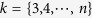 and 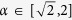.

*Proof.* When 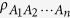 is a symmetric state, it is permutation invariant. Then, 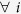, 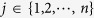 and *i* ≠ *j*, we have 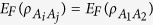 and


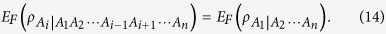


Combining with Eq. (11), we have


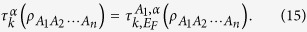


Moreover, according to Eq. (5), we have


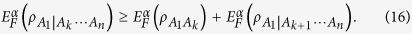


Then we can derive


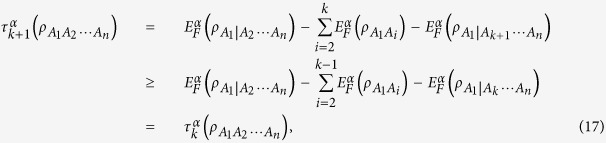


where the inequality holds because of Eq. (16). Therefore, the entanglement indicator 
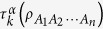
 is a monotonically increasing function of *k*.

In symmetrical quantum systems, the *k*-partite *n*-qubit monogamy relations of *α*EoF in Eq. (10) can be a monogamy equality (e.g., the corresponding results in the next subsection), and thus the corresponding entanglement indicator 
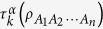
 can not work. However, we can choose an appropriate indicator


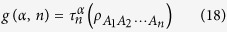


to represent a better entanglement indicator which comes from the following result.

**Theorem 3** For any *n*-qubit symmetric state 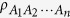, the entanglement indicator obeys the following relation


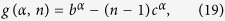


where 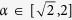, 
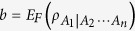
 and 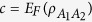. For any *n*, we have the following results

1. When *c* = 0, 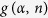 is a monotonically decreasing function of *α*.
2. When *c* > 0 and *b* < 1, 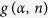 is a monotonically decreasing function of *α* if and only if

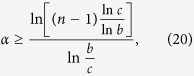


and 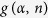 is a monotonically increasing function of *α* if and only if


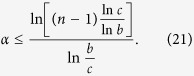


When *c* > 0 and *b* = 1, 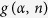 is also a monotonically increasing function of *α*.

*Proof.* From Eqs (10), (12) and (15), we have


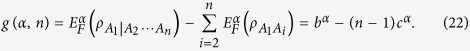


According to the definition of *b* and *c* and the monogamy inequality (5), we get 0 ≤ *c* < *b* ≤ 1.

For any *n*, we will analytically prove the two necessary and sufficient conditions.

1. When *c* = 0, we have 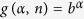. Because 0 < *b* ≤ 1, 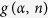 is a monotonically decreasing function of *α*.
2. When 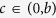, we have

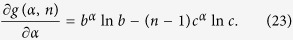


The monotonically decreasing property of 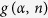 is satisfied if and only if the first-order partial derivative 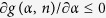, which is equivalent to Eq. (20).

Furthermore, the monotonically increasing property of 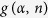 is satisfied if and only if the first-order partial derivative 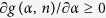, which is equivalent to Eq. (21).

From Theorem 3, we can obtain that the necessary and sufficient condition for the unit indicator is 
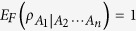
 and 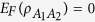. For any *n*-qubit symmetrical state, we can numerically compute the corresponding bounds to determine which is better, 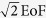 indicator or the 2 EoF, as follows:

After some deduction, we numerically obtain two bounds *N*_1_ and *N*_2_ with Eqs (20) and (21). When *n* ≥ *N*_1_, the 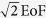 indicator is better than the 2 EoF indicator which comes from Eq. (20). The 2 EoF indicator is better than the 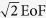 indicator when *n* ≤ *N*_2_, which comes from Eq. (21).

These results can be verified via two *n*-qubit symmetrical states in the next subsection.

### Analytical examples

We will investigate the above results on permutationally invariant states, which are the *W* state, the superposition of the *W* state and the Greenberger-Horne-Zeilinger (*GHZ*) state of *n* qubits respectively.

#### For the *W* state

In this part, we analyze the *n*-qubit *W* state which has the form


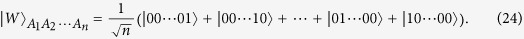


For this quantum state, the *n*-partite *n*-qubit monogamy relations of *α*th power of concurrence as shown in ref. 7 are saturated, and thus these concurrence-based entanglement indicators can not work. However, we will show that the *α*EoF-based indicator can be used to represent the entanglement in the *n*-partite *n*-qubit systems.

Using the symmetry of qubit permutations in the *W* state, 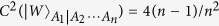, and 
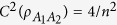
 17, we have


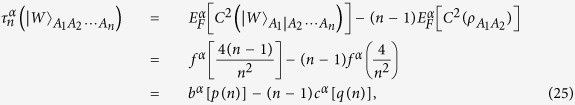


where 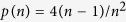 and 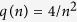. This set of 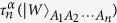 are positive since the *α*EoF is monogamous as shown in Eqs (5) and (10).

In order to study the properties of 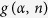, we firstly prove the function *M*(*n*), with


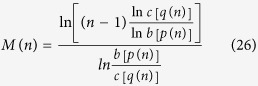


in Eqs (20) and (21), is a monotonically decreasing function of *n*. The details for illustrating the monotonic property are presented in Methods.

Let


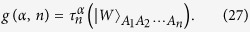


After some deduction, we can derive


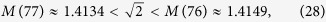


when 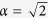. Thus, combining with the monotonically decreasing property of *M*(*n*), we prove that *α* ≥ *M*(*n*) when *n* ≥ 77, while *α* ≤ *M*(*n*) when *n* ≤ 76. When *α* = 2, we get


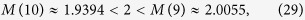


which means *α* ≥ *M*(*n*) when *n* ≥ 10, while *α* ≤ *M*(*n*) when *n* ≤ 9. Combining the above two inequations with Eqs (20) and (21), we obtain the two bounds 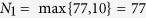 and 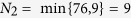. And, we know that 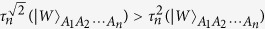 when *n* ≥ *N*_1_, and 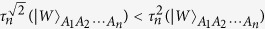 when *n* ≤ *N*_2_. Then we complete the proof that 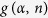 obeys these properties.

**Figure 1:**
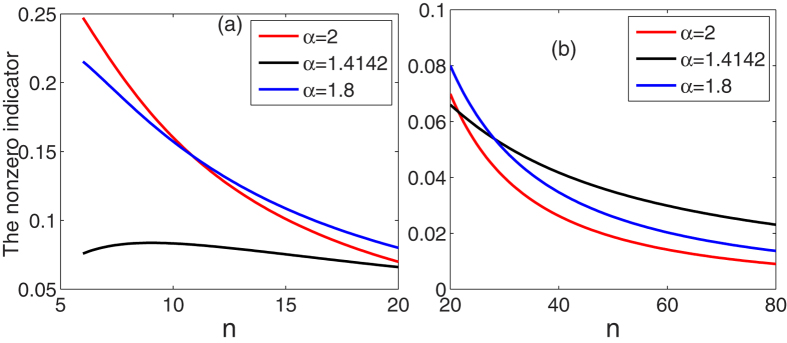
The multipartite entanglement indicators for the *W* state as functions of *n*, where 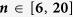 in (a) and 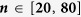 in (b).

In Fig. 1, we plot these indicators as functions of *n*, and then these properties can be verified from the figure. From the Fig. 1, we numerically find that 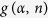 is a monotonically decreasing function of *n* when 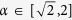 and *n* ≥ 10. How to exactly prove the result is an open problem.

These results still hold for symmetric *n*-qubit mixed states as shown in the next part.

#### For the superpositions of the *GHZ* state and the *W* state

When an *n*-qubit mixed state is a superpositions of the *GHZ* state and the *W* state, it has the form


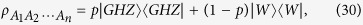


where 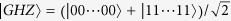 and 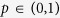. For *n* = 3, Lohmayer *et al.*^5^ found that, when 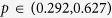, it is entangled but without two-qubit concurrence and three-tangle. It is still an unsolved problem^4^ of how to characterize the entanglement structure in this kind of states for large *n*.

In Eq. (18), the *n*-partite entanglement indicators have the forms


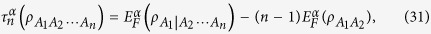


Then, the calculations of 
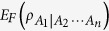
 and 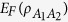 are key steps.

Any reduced two-qubit states of 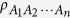 has the same form


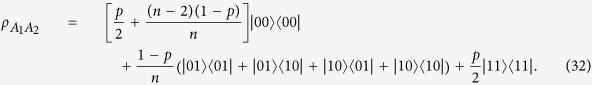


Using the effective method for calculating concurrence in ref. 15 and after some calculations, we have


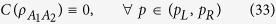


where *n* ≥ 6 and 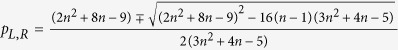. Then, according to Eq. (6), we obtain 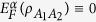.

In the following, we will calculate 
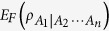
. Through introducing a system *B* which has the same state space as the composite system 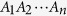, 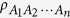 can be purified as


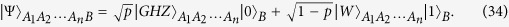


According to the Koashi-Winter formula^4^,^18^, the bipartite multiqubit EoF can be calculated by the purified state 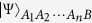, with 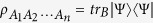,


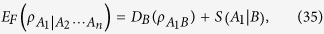


where 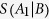 is the quantum conditional von Neumann entropy, and the quantum discord 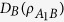 is defined as^13^


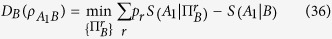


with the minimum running over all the positive operator-valued measures on the subsystem *B*. The details for proving Eq. (35) are presented in Methods. Chen *et al.*^19^ presented an effective method for choosing an optimal measurement over *B* and then calculating the quantum discord of two-qubit *X* states, which can be used to quantify the multipartite entanglement indicator in Eq. (19). After some analysis, we can obtain the optimal measurement for the quantum discord 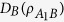 is *σ*_*z*_ when *n* ≥ 6 and 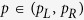. Then, after some deduction, we get


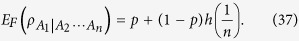


From Eqs (19), (31) and (33), the indicator has the form


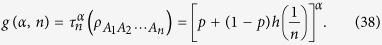


**Figure 2:**
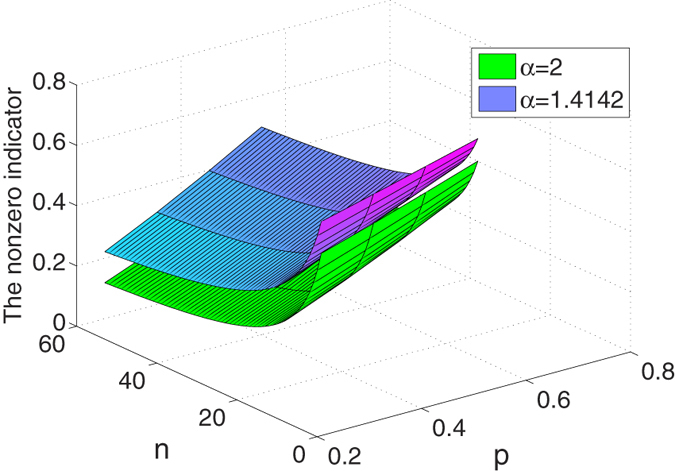
The multipartite entanglement indicators for the superposition state as functions of *n* and *p*, where 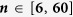, *α* = 2 and 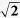 respectively.

The distribution of 
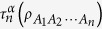
 has been shown in Fig. 2 for 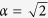 and *α* = 2 respectively. Furthermore, 
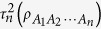
 and 
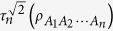
 have some properties as follows.

1. For any *α*, 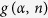 is a monotonically decreasing function of *n*. The monotonically decreasing property of 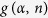 holds because the first-order partial derivative satisfies 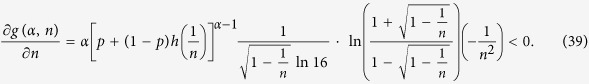


2. Combining with Theorem 3 and Eqs (33) and (38), we have 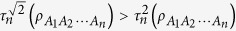.

From the above two properties, we know that the nonzero 
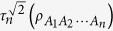
 can indicate the existence of the *n*-qubit entanglement. These results can also be understood as the fact that 
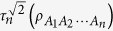
 can detect as many as possible *n*-qubit entangled states for large *n*.

## Conclusion

Entanglement monogamy is a fundamental property of multipartite entangled states. Based on our established monogamy relations Eq. (10), we obtain a set of useful tools for characterizing the multipartite entanglement not stored in pairs of the focus particle and the other subset of particles, which overcome some flaws of the concurrence. For any *n*-qubit symmetric state, we prove that the 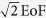 indicator work best when *n* is large enough, while the 2 EoF indicator works better than the 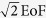 indicator for smaller *n*.

## Methods

### The monotonic property of the function in Eqs (20) and (21)

In order to determine the monotonic property of *M*(*n*), with


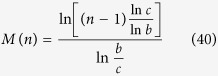


in Eqs (20) and (21), we analyze the sign of the first-order derivative *dM*(*n*)/*dn*.

After some deduction, we can obtain


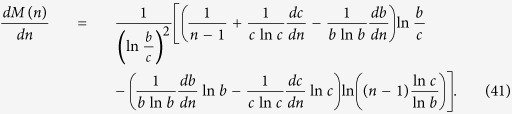


Then, *dM*(*n*)/*dn* < 0 when


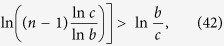


and


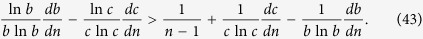


Eq. (42) holds if and only if


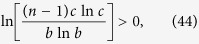


i.e.,


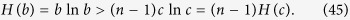


The inequality (45) holds because 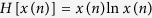 is a concave function of *n* with 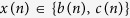.

Similarly, we have Eq. (43) holds when


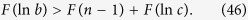


where


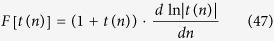


and then 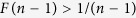. From ref. 9, we easily get that *dt*(*n*)/*dn* < 0 where 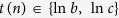.

In the following, we will prove Eq. (46). Let 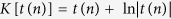, where 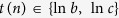. Using the definition of the partial derivative, it is not different to verify that 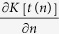, 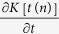, 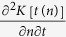 and 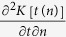 are all continuous functions. Combining with the exchange order theorem of two second-order mixed partial derivative, we have


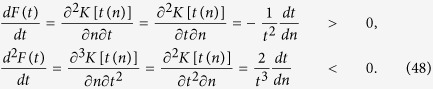


According to Eq. (47), we get that *F*(*t*) is monotonic and concave as a function of *t*.

Combining with Eq. (19), we have


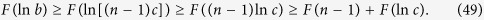


Here, the first inequality holds because *f* is a concave function of *n*, and the monotonically increasing property of *F*(*t*) in Eq. (48). The second inequality is satisfied because *F*(*t*) is a monotonically increasing function in Eq. (48) and ln *x* is a concave function of *x*. And the last inequality holds because *F*(*t*) is a concave function as proved in Eq. (48).

Then, we complete the proof that *M*(*n*) is a monotonically decreasing function of *n*.

### Proof of the Eq. (35) in the Main Text

Purification can be done for any state 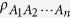, because we can introduce a system *B* which has the same state space as system 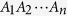 and define a pure state^20^ for the combined system


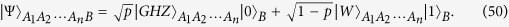


From ref. 21, we know


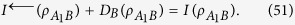


Combining with 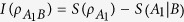, we can find that Eq. (35) is just Eq. (2) in ref. 17. More specifically,


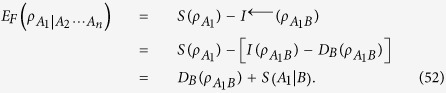


Then, we complete the proof of the Eq. (35) in the Main Text.

## Additional Information

**How to cite this article**: Liu, F. *et al.* Multipartite entanglement indicators based on monogamy relations of n-qubit symmetric states. *Sci. Rep.* **6**, 20302; doi: 10.1038/srep20302 (2016).
